# Network Pharmacology-Based Prediction and Verification of the Targets and Mechanism for Panax Notoginseng Saponins against Coronary Heart Disease

**DOI:** 10.1155/2019/6503752

**Published:** 2019-07-03

**Authors:** Yan Dong, Lian Duan, Heng-wen Chen, Yong-mei Liu, Yun Zhang, Jie Wang

**Affiliations:** Department of Cardiology, Guang'anmen Hospital, China Academy of Chinese Medical Sciences, Beijing, China

## Abstract

Coronary heart disease (CHD) is the worldwide leading cause for cardiovascular death. Panax notoginseng saponin (PNS), which is the main bioactive compound of panax notoginseng, has been generally accepted to exert a remarkable effect on CHD for a long time. However, to reveal the underlying treatment target and corresponding mechanism of PNS against CHD is still a substantial challenge. In this work, the targets and mechanism of PNS against CHD were successfully achieved by pharmacology-based prediction and experimental verification. 36 common targets were screened out through integrating the gene expression profile of CHD and the chemical-protein data of PNS. Then, two key nodes were further selected for verification by experiment after analyzing GO function, KEGG pathway, coexpression, and topology analysis. Results showed that PNS has protected the human umbilical vein endothelial cells from H_2_O_2_-induced oxidative stress by inhibiting early cell apoptosis via upregulating VEGFA mRNA expression. Therefore, our research has successfully pointed out one treatment target and apoptotic inhibition caused by PNS with method of integrating bioinformatics prediction and experimental verification, which has partially explained the pharmacological mechanism of PNS against CHD.

## 1. Introduction

Cardiovascular disease (CVD), as the leading reason in death worldwide, would increasingly result in over 23.6 million deaths by 2030 [1]. Coronary heart disease (CHD) is the most common cause of cardiovascular death, which accounts for about 44% [2]. 2018 AHA statistical data indicated that 16.5 million people over the age of 20 had CHD, and myocardial infarction (MI) occurred per 40 seconds approximately in America [2]. Moreover, silent MI or undiagnosed MI may account for almost 50% of the whole MIs [3]. This nonmanifestation MI is also an important risk factor for development of future heart failure [4]. Therefore, it could be speculated that the actual incidence of CHD is possibly higher than the reported data. Currently, the treatment strategy of CHD has achieved much progress involving new oral anticoagulant/antiplatelet drugs and drug eluting stent. However, recent treatment methods such as antiplatelet therapy, lipid regulation, and percutaneous coronary intervention (PCI) are still unsatisfactory for lots of patients worldwide due to difficulties in overcoming adverse effects. For example, as one cornerstone of CHD treatment, antiplatelet therapy encountered challenges of drug resistance [5, 6] and inappropriate use [7, 8] in clinical practice. Statins for lipid regulation sometimes induced the damage of liver and muscular cells [9]. Regarding PCI, there was increasing death rate observed from 2004 to 2014 [2]. As a result, it is noted that more effective and safer treatments for CHD are still urgently needed and continue to be challenging.

**Figure 1:**
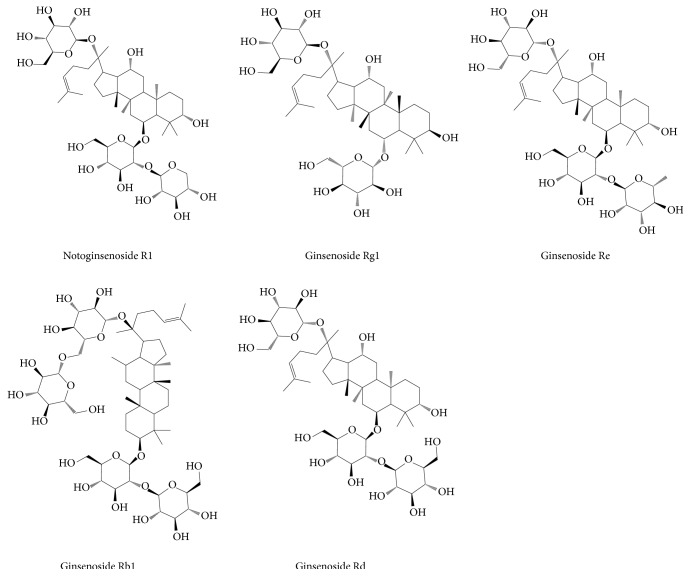
The chemical structures of five main compounds in PNS.

Panax notoginseng, as one of the traditional Chinese medicines (TCM) with effects of promoting blood circulation and stopping bleeding, has attracted enormous researches in the treatment of CVD. Panax notoginseng saponin (PNS) is the main bioactive compound in panax notoginseng, exerting cardiovascular protection. PNS mainly contains five compounds, namely, notoginsenoside R1, ginsenoside Rg1 (also called sanchinoside C1), ginsenoside Re, ginsenoside Rb1, and ginsenoside Rd (Figure 1), according to Pharmacopoeia of the People's Republic of China [10] and Hong Kong Chinese Materia Medica Standards [11]. Currently, PNS has been made into several preparations for CHD therapy. A meta-analysis revealed that panax notoginseng preparations are beneficial for unstable angina pectoris patients [12], possibly owing to the vital importance of PNS in antiplatelet aggregation [13], anti-inflammation [14], antioxidation [15], antiatherosclerotic [16], antiapoptosis [17], and lipid regulation [16]. However, essential compounds and specific targets of PNS are not clearly determined and the underlying mechanism against CHD remains poorly defined. Therefore, to reveal and understand the treatment targets and mechanism of PNS against CHD is a subject which is worthy of study.

**Figure 2:**
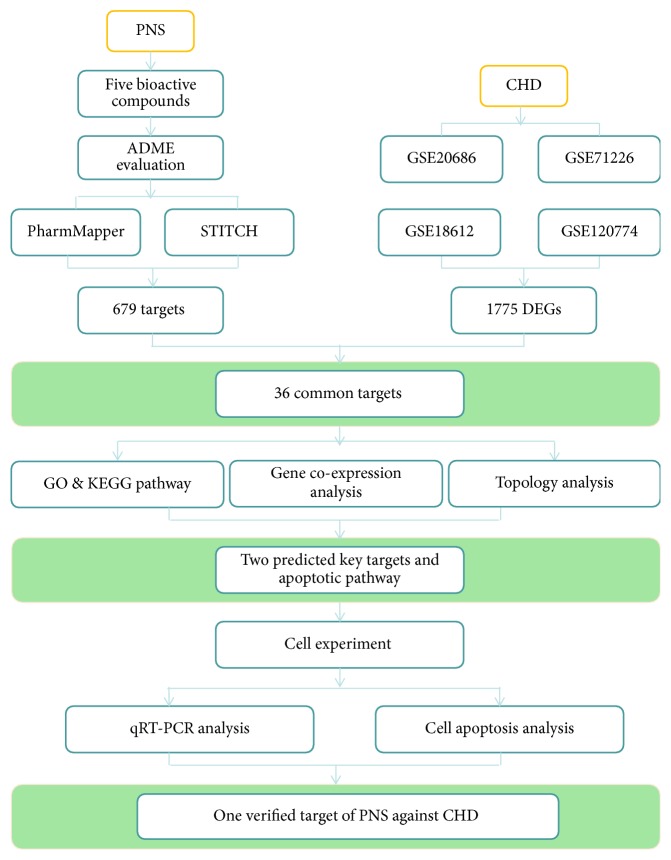
The whole framework of targets prediction and verification. ADME properties of PNS were first evaluated and then potential treatment targets of PNS against CHD were predicted through identifying common genes. Next, GO function, KEGG pathway, gene coexpression, and topology analysis were performed to predict key targets and their gene function and pathways. Finally, cell experiment was conducted to verify the selected targets and underlying mechanism.

Since network pharmacology is an effective approach for drug target prediction, it has been increasingly growing in popularity [18–20]. It could offer complementary support for new drug development and better understanding of complex mechanisms for drugs. Moreover, it can also provide systematic and holistic methods to deeply reveal the drug-gene-disease interactions which are especially suitable for understanding the multitarget, complementary, and synergic essence of TCM in molecular networks. Nevertheless, analysis of drug target based on network pharmacology is still in its infancy stage [21]. Predicted targets still need further confirmation from experiments. In this study, we predicted and verified the targets and mechanism for PNS against CHD through coupling the network pharmacology approach and experimental method. By integrating bioinformation from databases involving TCMSP, PharmMapper, STITCH, Gene Expression Omnibus (GEO) DataSets, WebGestalt, and GeneMANIA, key targets and corresponding mechanism of PNS against CHD were predicted. Then, cell experiment was applied to verify two key genes. As a result, one verified gene was identified as the treatment target of PNS against CHD, and the potential mechanism was regulation of apoptosis. The whole flow chart of our network prediction and experimental verification was shown in Figure 2.

## 2. Materials and Methods

### 2.1. Evaluation of ADME-Related Properties for PNS

PNS mainly contains notoginsenoside R1, ginsenoside Rg1, ginsenoside Re, ginsenoside Rb1, and ginsenoside Rd. The last compound is often exhibited as the metabolite, ginsenoside Rd qt. In order to assess druggable characters of the above five compounds, ADME (abbreviation of absorption, distribution, metabolism, and excretion) properties are evaluated by using TCMSP server (http://lsp.nwu.edu.cn/tcmsp.php). Particularly, important parameters of oral bioavailability (OB), drug-likeness (DL), molecular weight (MW), blood-brain barrier (BBB), intestinal epithelial permeability (Caco-2), fractional negative surface area (FASA-), the polar surface area (TPSA), and RBN [22] are used. Among all the descriptive parameters, OB and DL are the most two important indicators [23, 24] to assess and identify candidate compounds or drugs. OB is closely related to drug absorption and delivery of an orally administered dose. Drugs with high OB value mean they are more likely to reach the potential targets and can be therapeutic agents. DL can be used to estimate the druggability of a drug qualitatively, which helps to optimize pharmacokinetic and pharmaceutical properties. Commonly, OB*⩾*30% and DL*⩾*0.18 are suggested as drug screening criteria in the traditional Chinese herbs [22]. In the TCMSP server, OB value is calculated by an in-house model OBioavail1.1 [22]. Besides, the Tanimoto coefficient [25] is used to assess DL indexes of each compound in PNS. The formula is shown as follows [26]:

(1) $$\begin{array}{c} T\left(\;\alpha ,\beta \;\right)=\frac{\alpha \times \beta }{\alpha^{2}+\beta^{2}-\alpha \times \beta } \end{array}$$

### 2.2. Targets Prediction of PNS against CHD

Pharmmapper (http://lilab-ecust.cn/pharmmapper/) and STITCH (http://stitch.embl.de/) were used to identify targets related to PNS in the study. Pharmmapper is a reverse docking server which identifies potential protein targets for small molecule compounds via a pharmacophore mapping approach. By calculating the fit scores and z'-scores (normalized fit scores) of a compound with different targets respectively, the correlation degree between the query compound and its targets are predicted. Generally, large positive z'-score indicates high significance [27], while STITCH provides a collection of protein-chemical interactions that integrates many sources of experimental and manually curated lines of evidence with text-mining information and interaction predictions [28, 29]. It calculates a score for each pair of protein-chemical interaction. Usually, interactions with score *⩾*0.4 are considered as medium confidence and those with the score*⩾*0.7 are high confidence [30].

In addition, gene expression series of CHD were downloaded from GEO DataSets [31] (http://www.ncbi.nlm.nih.gov/geo/). Four researches with GSE20686, GSE71226, GSE18612, and GSE120774 accession numbers were offered in the platform of GPL4133, GPL570, and GPL6244, respectively. The expression profiles were all received by array. After unifying different gene names as Gene Symbol by UniProtKB (http://www.uniprot.org), the differential expression genes (DEGs) of CHD were obtained. Furthermore, Venn diagram (http://bioinformatics.psb.ugent.be/webtools/Venn/) was drawn to identify the intersection nodes of PNS and CHD. Finally, 36 common targets were predicted.

### 2.3. GO Function, KEGG Pathway, and Key Targets Analysis

GO function and KEGG pathway of 36 common targets were analyzed with WebGestalt (http://www.webgestalt.org/). Then, coexpression network of common targets was established by GeneMANIA (http://pages.genemania.org/) which was usually applied to generate hypotheses about gene function, analyze gene lists, and prioritize genes for functional assays [32]. In addition, topology analysis was conducted by CentiScape and MCODE to calculate the degree centrality and extract subnetwork, in order to identify key nodes in the coexpression network. Ultimately, two key targets which participate in apoptotic signaling pathway were selected to perform further cell experiment.

### 2.4. Experimental Materials and Cell Culture

Human umbilical vein endothelial cells (HUVECs) were purchased from Bei Na Chuanglian Biotechnology Research Institute, Beijing, China. PNS was purchased from National Institutes for Food and Drug Control, Beijing, China. PNS was dissolved with Phosphate Buffered Saline (1X) (Hyclone, Lot AC12557265) before experiment. Dulbecco's modified Eagle's medium (DMEM, Hyclone, Lot AD19822332) supplemented with 10% fetal bovine serum (Gibco, Lot 1739463) and 1% penicillin-streptomycin (Hyclone, Lot J160016) was used to culture HUVECs. The cultures were maintained in a humidified atmosphere containing 5% CO_2_ at 37°C. Then cells were inoculated onto 6-well plates to perform experiments and each group had 3 samples. Treatment groups were pretreated with 30 *μ*g/mL PNS for 21 h and then added into 100 *μ*mol·L^−1^ H_2_O_2_ for 3 h. In contrast, model groups were only treated with 100 *μ*mol·L^−1^ H_2_O_2_ in the last 3 h. Cells in control groups were cultured in medium added with equal volume of DMEM.

### 2.5. RNA Isolation and Real-Time PCR Analysis

Total RNAs were extracted by using TRIzol reagent (Ambion, Lot 124006). Reverse transcript PCR was performed by FastQuant RT Kit (TianGen Biotech co., ltd, N2828, Beijing, China). Real-time quantitative PCR was conducted by PowerUP SYBR Green PCR Master Mix (Applied Biosystems, Lot 1710043) on 7900HT fluorescence quantitative PCR machine (Applied Biosystems). The primers of the two target genes and reference gene were as follows: vascular endothelial growth factor A (VEGFA) (forward: 5′-ACGAACGTACTTGCAGATGTG-3′; reverse: 5′-TTCTGTCGATGGTGATGGTGT-3′); B-cell lymphoma 2-related protein A1 (BCL2A1) (forward: 5′-GCACAATCACACACCTATGCT-3′; reverse: 5′-TGTGTTGGCAATCGTTTCCAT-3′); *β*-actin (forward: 5′-GAGACCTTCAACACCCCAGCC-3′; reverse: 5′-AATGTCACGCACGATTTCCC-3′). The relative mRNA expressions of VEGFA and BCL2A1 were calculated by 2^–ΔΔCT^  (ΔCt = Ct value of VEGFA or BCL2A1 minus Ct value of *β*-actin; ΔΔCt = ΔCt of the treatment group minus ΔCt of the treatment group) and fold change (FC). In order to ensure the quantitative accuracy, the experiments were performed in triplicate.

### 2.6. Cell Apoptosis Analysis

HUVECs were seeded in 6-well plates (1×10^6^ cells/well) and incubated with 30 *μ*g/mL PNS and/or 100 *μ*mol·L^−1^ H_2_O_2_. After the culture for 24 h, cells were harvested and washed with cold PBS twice. Then cells were resuspended in 1×Binding Buffer and stained with FITC Annexin V and Propidium Iodide (PI) supplied in the FITC Annexin V Apoptosis Detection kit I (BD Pharmingen, USA). Next, the cells were incubated for 15 min at RT (25°C) in the dark. Finally, 1×Binding Buffer was added to cells and those cells were analyzed by a flow cytometer (BD FACSCalibur, USA) within 1 h. All experiments were performed in triplicate.

### 2.7. Statistical Analysis

The quantitative data were presented as mean±standard deviation (±SD). One-way analysis of variance (ANOVA) was applied to compare multiple means. When the variance was equal, the least-significant difference (LSD) method was used. Dunnett's T3 test was conducted when the variance was not equal. Significance was defined as P<0.05. All statistical analysis was performed with SPSS 17.0 and data were presented as two decimals. In addition, all statistical column charts were drawn with Origin 8.0.

## 3. Results

### 3.1. ADME-Related Properties of PNS

All compounds of PNS showed superior DL value, except for ginsenoside Rb1. Particularly, ginsenoside Rg1 and ginsenoside Rd qt had the DL value above 0.18, which indicated that they presented excellent druggability such as better solubility and chemical stability [26]. Similarly, their OB indices which were estimated to be 10.04% and 12.23%, respectively, demonstrated that they can be efficiently absorbed to systemic circulation, compared with other three compounds. As for MW, TPSA, and RBN parameters, ginsenoside Rd qt showed better properties than others as well (see Table 1). Therefore, it could be seen that ginsenoside Rd qt was more druggable and may be the main bioactive compound in oral management.

### 3.2. Identification of Potential Targets for PNS

**Figure 3:**
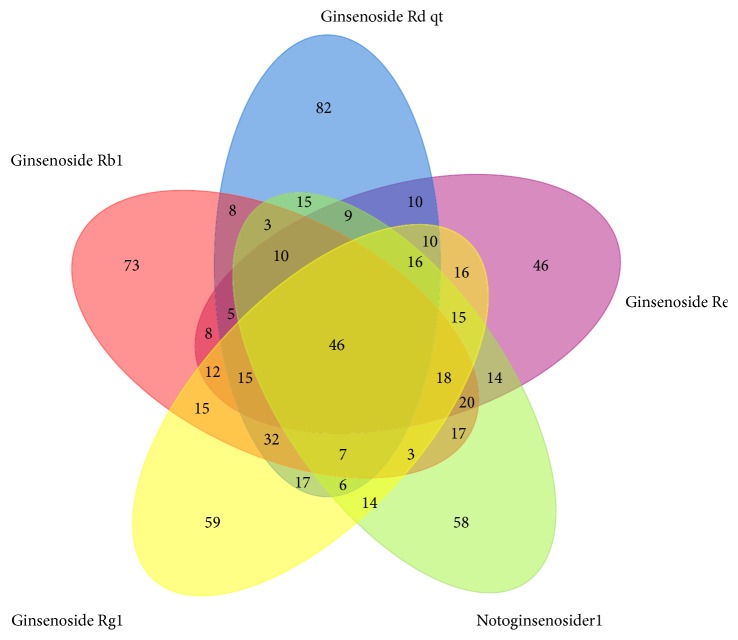
Common targets of five compounds in PNS.

Potential targets were predicted by combination of Pharmmapper and STITCH servers. Firstly, the Tripos mol2 files of 5 compounds from TCMSP server were put into Pharmmapper, respectively. According to the z'-score, the top 300 potential targets of each compound were obtained (see attachment 1). Then, chemical names of notoginsenoside R1, ginsenoside Rg1, ginsenoside Re, ginsenoside Rb1, and ginsenoside Rd were put into STITCH to match their potential targets, with the minimum required interaction score being 0.4 (medium confidence). Correspondingly, 24 different targets involving NOS3, NR3C1, PRKAA1, STK11, MMP9, RB1, VEGFA, CHRNA2, CHRNA4, CHRNA7, RHAG, CHRFAM7A, RBL2, BDNF, AKT1, CASP3, CREB1, MAPK11, MAPK14, HBEGF, NFE2L2, CREM, CYCS, and TRPM7 were thereby predicted. Furthermore, after unifying different gene names as Gene Symbol by UniProtKB, 679 different targets were identified totally, among which 46 common targets were shared by all the compounds (Figure 3).

### 3.3. Common Targets of PNS and CHD

**Figure 4:**
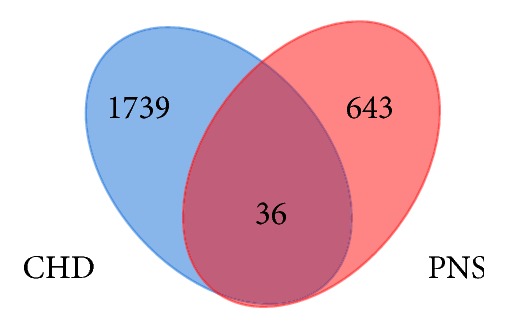
Common targets of CHD and PNS.

CHD-related genes were searched in GEO database and the series (GSE20686, GSE71226, GSE18612, and GSE120774) were selected. The former two contained DEGs of 399 samples totally from peripheral blood, while the latter two involved DEGs of 49 samples totally from epicardial and subcutaneous adipose tissue which were closely related to coronary atherosclerosis [33–35]. CHD is diagnosed when the luminal stenosis*⩾*50% in at least one major vessel. Therefore, patients who met the above diagnostic criteria were assigned to the CHD group, and the rest belonged to non-CHD group, when analyzing GSE20686 and GSE71226 data. Regarding GSE18612 and GSE120774, patients who underwent cardiac surgery for coronary artery disease were assigned to the CHD group; and those undergoing cardiac surgery for other reasons were considered as non-CHD group. By comparing CHD and non-CHD groups with GEO2R, there were 1775 DEGs finally identified in the above four series with selection criteria of FC*⩾*1.5 and P<0.05. Moreover, further comparison between 1775 CHD-related genes and 679 PNS targets identified 36 common genes that may become the predicted treatment targets of PNS against CHD (Figure 4).

### 3.4. GO Function and KEGG Pathway Analysis of 36 Common Targets

**Figure 5:**
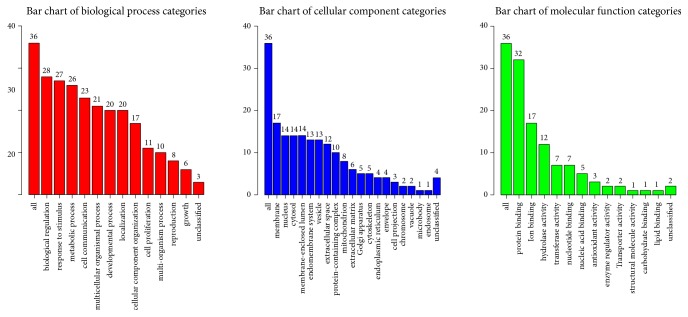
GO enrichment of 36 common genes. Different categories of biological process, cellular component, and molecular function were represented by a red, blue, and green bar, respectively. The height of the bar represented the number of genes observed in the category.

To investigate the 36 common genes of PNS and CHD, GO function and KEGG pathway were analyzed with WebGestalt. GO enrichment was analyzed from the perspective of biological process, cellular component, and molecular function. It was noted that the common genes were mainly responsible for protein binding, biological regulation, response to stimulus, and metabolic process (Figure 5). Importantly, two apoptotic signaling pathways have been enriched in the top 10 GO functions (as shown in Table 2).

**Figure 6:**
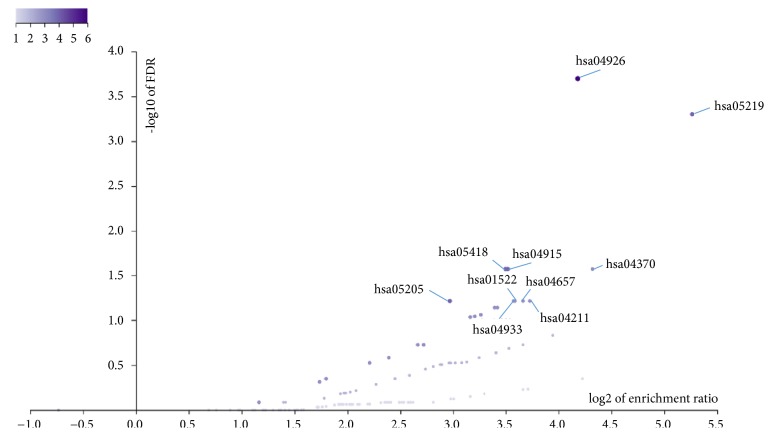
KEGG enrichment of 36 common genes. Each purple point represented a pathway. The darker the color of a point, the more the enriched genes contained in the pathway.

Additionally, KEGG pathways of the common genes were analyzed and shown in Figure 6. Among all the pathways, hsa04926, hsa05219, hsa04915, hsa05418, hsa05205, hsa04370, hsa04211, hsa04657, hsa01522, and hsa04933 enriched the most genes and ranked the top 10 levels (Table 3). Particularly, relaxin signaling pathway, fluid shear stress and atherosclerosis, VEGF signaling pathway, and IL-17 signaling pathway were reportedly related to CHD [36–38].

### 3.5. Coexpression and Topology Analysis of 36 Common Targets

**Figure 7:**
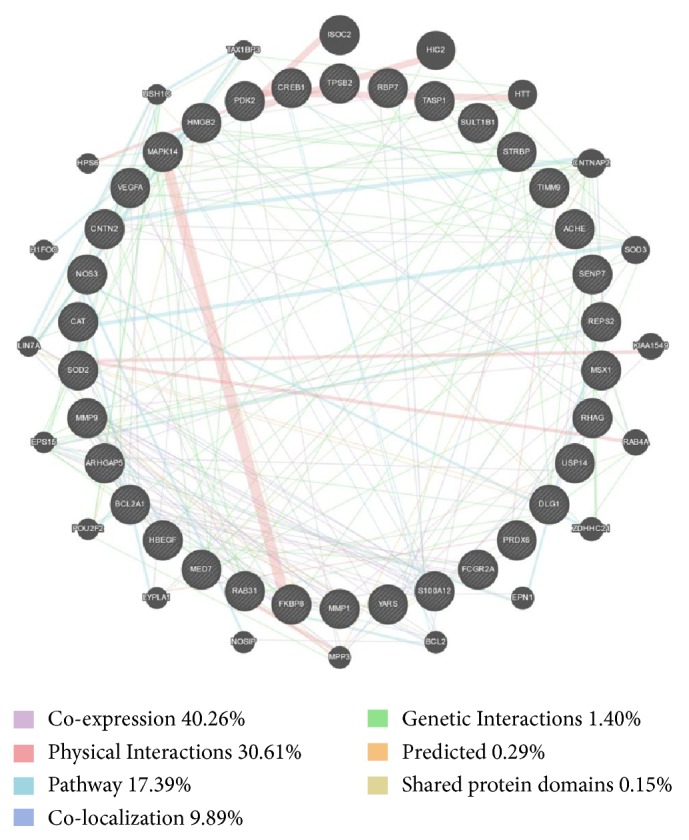
The coexpression network of common targets for PNS against CHD. Circle nodes mean genes, and edges with different connecting colour represent different correlations. Genes in black circles with white stripes were submitted as query terms, while pure black circles were genes associated with query terms. Genes with larger circle indicate more correlations with other genes in the network.

To further evaluate coexpression characteristics of 36 common targets, GeneMANIA was used to analyze their interactions among each other and with other related genes. The results showed that 34 common targets involving PDK2, DLG1, ACHE, TASP1, TIMM9, ARHGAP5, MMP9, USP14, FKBP8, RHAG, SOD2, VEGFA, HBEGF, PRDX6, CREB1, CAT, YARS, SENP7, BCL2A1, FCGR2A, MED7, RBP7, MSX1, S100A12, HMGB2, STRBP, RAB31, REPS2, SULT1B1, CNTN2, MMP1, TPSB2, MAPK14, and NOS3 were recognized in GeneMANIA. Through complex interactions involving coexpression, physical interactions, pathways, and colocalization, those 34 nodes established a coexpression network with other 20 nodes in GeneMANIA (Figure 7).

**Figure 8:**
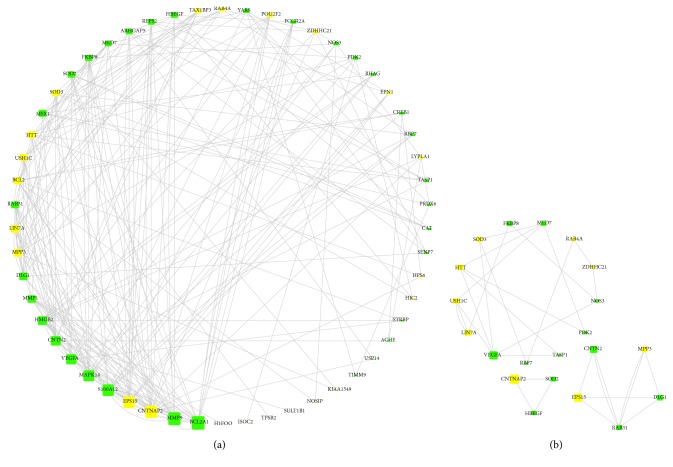
Topology analysis of the coexpression network. Figure (a) was the analysis of degree centrality and Figure (b) was k-core decomposition. Green rectangles were common genes which meant query terms. Yellow rectangles were genes associated with query terms in the above coexpression network. The gray edges stand for the interactions among different genes. Gene with more edges had larger rectangle size and the size corresponded to its degree in the coexpression network. Larger size indicated higher degree. The three subnetworks in Figure (b) were all extracted from Figure (a).

In addition, topology analysis was performed to identify key nodes in the coexpression network, involving calculation of degree centrality and k-core decomposition by CentiScape and MCODE, respectively, in Cytoscape 3.6.1. Degree centrality refers to the degree to which a node in a network is associated with all other nodes. Node with higher degree usually means more interaction edges and more significance in the network. The k-core of a network is defined as the largest subnetwork where every node has at least k links. Commonly, the extracted subnetwork is considered to be more stable and often used for hub nodes screening [39]. In this study, we set the parameters of degree>15 and k-core=2 to help identify key nodes in the coexpression network. Results indicated that common genes involving BCL2A1, MMP9, MAPK14, S100A12, and VEGFA have degree above 15 (Figure 8(a)). Furthermore, three subnetworks were extracted from the coexpression network by k-core decomposition (Figure 8(b)). Interestingly, it is shown in our analysis that VEGFA also constituted one part of a subnetwork, showing more possibility as a key node in the whole network. More importantly, VEGFA participates in regulation of apoptosis and BCL2A1 is a well-known antiapoptotic gene [40, 41]. As a result, we selected the two key nodes of VEGFA and BCL2A1 as potential targets for next experimental verification. Additionally, cell apoptosis was also observed to reveal VEGFA and BCL2A1-involved pathway in the treatment.

### 3.6. PNS Upregulated VEGFA mRNA Expression in H_2_O_2_-Treated HUVECs

**Figure 9:**
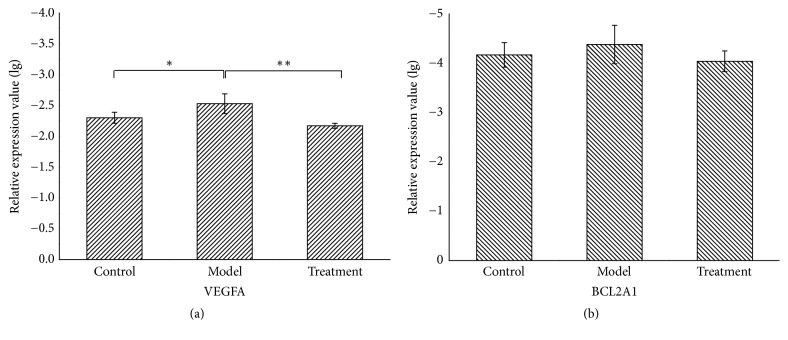
Statistical analysis of VEGFA (a) and BCL2A1 (b) expression in the control, model, and treatment groups. Relative expressions of VEGFA and BCL2A1 in each group are presented as lg(2^−△CT^) value in the above figures. All results are presented as mean ± SD (n = 3). Significance is defined as p< 0.05 (*∗*p < 0.05; *∗∗*p < 0.01).

To verify the predicted targets of VEGFA and BCL2A1 in PNS against CHD, 100 *μ*mol·L^−1^ H_2_O_2_-induced HUVECs injury was performed as the oxidative stress model in cardiovascular diseases [42, 43]. In our experiment, the mRNA expressions of VEGFA and BCL2A1 were detected by real-time PCR in control, model, and treatment groups, respectively. According to the analyzing method of real-time PCR data [44], we performed the calculation of 2^−△△CT^ values and analyzed corresponding FC and expression level. The detail values were described in Table 4. Furthermore, statistical differences of relative expression of VEGFA and BCL2A1 in each group were also analyzed, respectively. Results showed that H_2_O_2_ significantly reduced VEGFA expression compared with the control group (P=0.041); moreover, PNS promoted VEGFA markedly compared to model group (P=0.007) (Figure 9(a)). With respect to BCL2A1, the mRNA expression of BCL2A1 had the trend to be downregulated by H_2_O_2_ and upregulated by PNS, but current results showed no statistical significance (P>0.05) (Figure 9(b)).

### 3.7. PNS Inhibited Early Apoptosis in H_2_O_2_-Treated HUVECs

**Figure 10:**
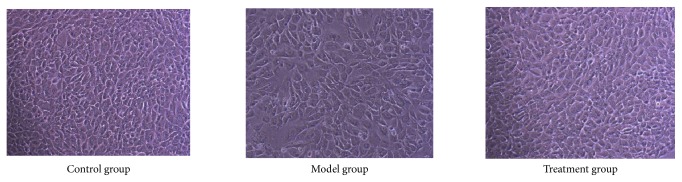
The number and morphology of HUVECs under 10x microscopes in different groups. Treatment group was pretreated with 30ug/ml PNS for 21h and then treated with 100 *μ*mol·L^−1^ H_2_O_2_ for 3h. Model group was only treated with 100 *μ*mol·L^−1^ H_2_O_2_ in the late 3h. Control group was cultured in medium added with equal volume of DMEM.

**Figure 11:**
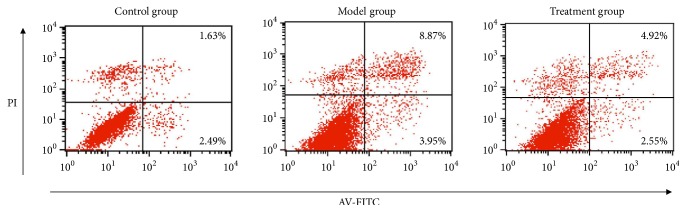
Apoptosis analysis of HUVECs in different groups. Intervention methods of control, model, and treatment groups were the same as those in Figure 10. The first quadrant represents cell death that is not related to apoptosis. The second quadrant represents the late phase of cell apoptosis. The third quadrant represents normal cell. The fourth quadrant represents the early phase of cell apoptosis.

**Figure 12:**
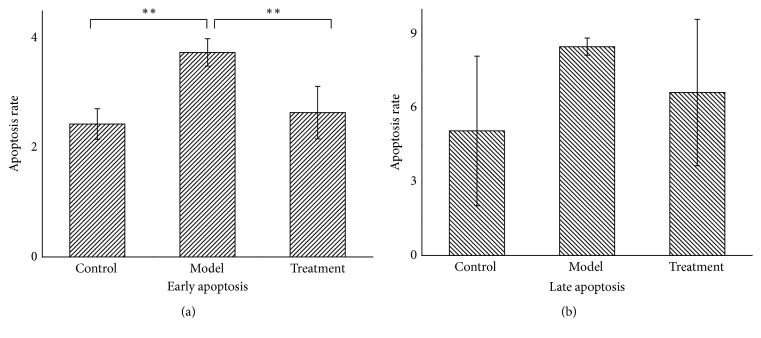
The early (a) and late (b) apoptosis rate of HUVECs in different groups. Results were presented as mean ± SD (n = 3). Significance was defined as p < 0.05 (*∗*p < 0.05; *∗∗*p < 0.01).

Previous reports have pointed out that VEGFA can participate in suppression of cell apoptosis [45, 46]. Thus, we further carried out apoptosis analysis to find out whether VEGFA overexpression induced by PNS could inhibit cell apoptosis in HUVECs. Our results showed that HUVECs in the model group were obviously reduced and disorderly arranged, while treatment group cells grew well compared with the model group (Figure 10). Apoptosis analysis further indicated that H_2_O_2_ induced cell apoptosis, but PNS pretreatment could protect cell from apoptosis (Figure 11). Particularly, the early apoptosis rate in model group was significantly higher than that in control group (P=0.004, Figure 12(a)). In contrast, PNS pretreatment markedly relieved H_2_O_2_-induced damages and protected HUVECs from early apoptosis when compared with model group (P=0.009, Figure 12(a)). However, no significant difference existed in the late apoptosis, although declined rates were also observed in treatment group (Figure 12(b)). These results suggested that PNS inhibited HUVECs apoptosis mainly ascribing to the suppression of early phase.

## 4. Discussion

In this study, we revealed that Ginsenoside Rd qt had better pharmacokinetics properties than the other four compounds in PNS. Thus, Ginsenoside Rd qt may be the main bioactive compound that exerted protective effect in the treatment of CHD. Notably, all compounds had relatively low OB value compared with the recommended screening criteria by TCMSP server [22], which indicated that PNS was not suitable for oral administration directly. More preparations involving injection [12, 47, 48] and spray [49] were therefore widely applied in practice or studied for new drug development. Although PNS has shown positive druggability and efficacy in clinical application, current researches about its specific targets and mechanism against CHD are still insufficient. Targets prediction based on network pharmacology have been reported [50, 51], but those predicted results are not convincing enough. Experimental verification is further needed to confirm the authenticity and reliability. As a result, we combined network pharmacology and cell experiment to predict and verify target and underlying mechanism of PNS in the treatment of CHD in our study. The results showed that VEGFA and BCL2A1 had great potential as target genes. However, only VEGFA was proved to be able to act as the target and participate in inhibition of apoptosis by cell experiment. Previous studies reported consistent results, indicating that VEGFA was an important protective factor in apoptosis of cardiomyocytes [45] and microvascular endothelial cells [46]. Moreover, VEGFA also took part in lncRNA NEAT1/miR-377/VEGFA axis to gain the protective effect in cell apoptosis [52]. It shows that VEGFA overexpression induced by PNS which inhibits cell apoptosis is reasonable. Notably, the inhibitory effect focused on the early apoptosis, rather than late phase. Therefore, our study revealed that PNS could upregulate the mRNA expression of VEGFA to participate in the suppression of apoptosis in early phase.

VEGFA was identified as a treatment target of PNS against CHD by integrating bioinformatics prediction and experimental verification. But beyond that, some other nodes in the coexpression network may also play important roles in the treatment of CHD, especially for MMP9 and MAPK14. A meta-analysis indicated that MMP9-1562C/T polymorphism is markedly associated with CHD [53]. Thus, the circulating level of MMP9 was upregulated in coronary artery disease, which may act as a risk factor for evaluating the development and severity of disease [54]. A mechanistic study further revealed that MMP-9 activation could promote cell migration to stimulate angiogenesis and preserve cardiac function [55]. As for MAPK14, also called p38MAPK *α*, it played negative role in expression of vascular smooth muscle cell (VSMC) contractile genes and differentiation of VSMC [56]. Besides, it promoted platelet activation and served as a potential indicator of highly thrombotic lesions and no-reflow after myocardial infarction [57]. As a result, experimental verification for more potential targets needs to be performed in the future. In addition, because of the fact that the expression level of mRNA and protein may be inconsistent after treatment [58], the detection of the protein expression from VEGFA should be carried out by Western blot assay in subsequent research. Furthermore, there have been some reports [46, 52] about the modulation of VEGFA by miRNA and lncRNA. Therefore, to reveal the regulatory mechanism of PNS against CHD based on lncRNA-miRNA-VEGFA axis in the apoptosis pathway is also a subject which is worthy of exploration.

## 5. Conclusion

In conclusion, our study predicted and verified that PNS can promote mRNA expression of VEGFA for the suppression of early apoptosis in vascular endothelial cells to exert curative effect for CHD. The results helped partially explain the pharmacological mechanism of PNS against CHD. Moreover, our research provided an integrated method for discovery of drug targets and corresponding mechanism.

## Figures and Tables

**Table 1:** ADME-related properties of five main compounds in PNS.

Mol ID	Molecule Name	OB (%)	DL	MW	BBB	Caco-2	FASA-	TPSA	RBN
MOL007487	Notoginsenoside R1	5.43	0.13	933.27	-4.15	-3.01	0.22	298.14	12
MOL005341	Ginsenoside Rg1	10.04	0.28	801.14	-3.50	-2.27	0.24	239.22	10
MOL005338	Ginsenoside Re	4.27	0.12	947.3	-4.39	-3.2	0.25	298.14	12
MOL007476	Ginsenoside Rb1	6.29	0.04	1,109.46	-4.95	-3.72	0.23	377.29	16
MOL007480	Ginsenoside Rd qt	12.23	0.77	460.82	-0.01	0.65	0.24	60.69	4

**Table 2:** Top 10 GO functions of common genes in PNS against CHD.

Geneset	Description	Count	P-Value	FDR	Ratio
GO:0002446	neutrophil mediated immunity	7	0.000074	0.024778	6.5312
GO:0036230	granulocyte activation	7	0.000077	0.024778	6.4789
GO:0030099	myeloid cell differentiation	6	0.000140	0.024778	7.3264
GO:2001233	regulation of apoptotic signaling pathway	6	0.000152	0.024778	7.2122
GO:0006979	response to oxidative stress	6	0.000280	0.027615	6.4424
GO:0048872	homeostasis of number of cells	5	0.000166	0.024778	9.4445
GO:0072593	reactive oxygen species metabolic process	5	0.000204	0.024778	9.0387
GO:0090130	tissue migration	5	0.000325	0.027615	8.1763
GO:0097193	intrinsic apoptotic signaling pathway	5	0.000335	0.027615	8.119
GO:0042737	drug catabolic process	4	0.000200	0.024778	13.611

**Table 3:** Top 10 KEGG pathways of common genes in PNS against CHD.

Geneset	Description	Count	P-Value	FDR	Ratio
hsa04926	Relaxin signaling pathway	6	0.000001	0.000182	18.143
hsa05219	Bladder cancer	4	0.000003	0.000465	38.352
hsa04915	Estrogen signaling pathway	4	0.000300	0.027039	11.477
hsa05418	Fluid shear stress and atherosclerosis	4	0.000400	0.027039	11.312
hsa05205	Proteoglycans in cancer	4	0.001400	0.061165	7.823
hsa04370	VEGF signaling pathway	3	0.000400	0.027039	19.988
hsa04211	Longevity regulating pathway	3	0.001400	0.061165	13.251
hsa04657	IL-17 signaling pathway	3	0.001600	0.061165	12.681
hsa01522	Endocrine resistance	3	0.001800	0.061165	12.034
hsa04933	AGE-RAGE signaling pathway in diabetic complications	3	0.001900	0.061165	11.912

**Table 4:** Relative mRNA expression values of the two genes.

Gene	Group comparison	2^−△△CT^ value	FC value	P value	Expression
VEGFA	Model vs Control	0.62	-1.61	0.041	Downregulation
	Treatment vs Model	2.28	2.28	0.007	Upregulation

BCL2A1	Model vs Control	0.81	-1.23	>0.05	Downregulation
	Treatment vs Model	2.40	2.40	>0.05	Upregulation

## Data Availability

The data used to support the findings of this study are included within the article.

## Supplementary Materials

Supplementary Materials The supplementary materials contain 5 files which are the top 300 potential targets of each compound predicted from Pharmmapper Server.
